# Normal Echocardiographic Reference Values of the Right Ventricular to Left Ventricular Endsystolic Diameter Ratio and the Left Ventricular Endsystolic Eccentricity Index in Healthy Children and in Children With Pulmonary Hypertension

**DOI:** 10.3389/fcvm.2022.950765

**Published:** 2022-07-14

**Authors:** Sabrina Schweintzger, Stefan Kurath-Koller, Ante Burmas, Gernot Grangl, Andrea Fandl, Nathalie Noessler, Alexander Avian, Andreas Gamillscheg, Philippe Chouvarine, Georg Hansmann, Martin Koestenberger

**Affiliations:** ^1^Division of Pediatric Cardiology, Department of Pediatrics, Medical University Graz, Graz, Austria; ^2^Institute for Medical Informatics, Statistics and Documentation, Medical University of Graz, Graz, Austria; ^3^Department of Pediatric Cardiology and Critical Care, Hannover Medical School, Hanover, Germany; ^4^European Pediatric Pulmonary Vascular Disease Network, Berlin, Germany

**Keywords:** normative values, pediatric, endsystolic right to left ventricular ratios, left ventricular endsystolic eccentricity index, pulmonary hypertension

## Abstract

**Background:**

An accurate assessment of the right and left ventricle and their interaction is important in pediatric pulmonary hypertension (PH). Our objective was to provide normal reference values for the right ventricular to left ventricular endsystolic (RV/LVes) ratio and the LV endsystolic eccentricity index (LVes EI) in healthy children and in children with PH.

**Methods:**

We conducted an echocardiographic study in 769 healthy children (median age: 3.36 years; range: 1 day—18 years) and validated abnormal values in 44 children with PH (median age: 2.1 years; range: 0.1 months—17.7 years). We determined the effects of gender, age, body length, body weight, and body surface area (BSA) on RV/LVes ratio and LVes EI values. The RV/LVes ratio and LVes EI were measured from the parasternal short axis view between papillary muscle from the endocardial to endocardial surfaces.

**Results:**

Both, the RV/LVes ratio and the LVes EI were highly age-dependent: (i) neonates RV/LVes ratio [median 0.83 (range 0.53–1.37)], LVes EI [1.21 (0.92–1.45)]; (ii) 12–24 months old: RV/LVes ratio: [0.55 (0.35–0.80)], LVes EI: [1.0 (0.88–1.13)]; iii) 18th year of life RV/LVes ratio: [0.53 (0.32–0.74)], LVes EI: [1.0 (0.97–1.07)]. Healthy neonates had high LVes EI and RV/LVes ratios, both gradually decreased within the first year of life and until BSA values of about 0.5 m^2^, body weight to about 15 kg and body length to about 75 cm, but were almost constant thereafter. Children (>1 year) and adolescents with PH had significantly higher RV/LVes ratio (no PH: median 0.55, IQR 0.49–0.60; PH: 1.02, 0.87–1.26; *p* < 0.001) and higher LVes EI values (no PH: 1.00, 0.98–1.00; PH: 1.53, 1.26–1.71; *p* < 0.001) compared to those without PH. To predict the presence of PH in children > 1 year, we found the following best cutoff values: RV/LVes ratio ≥ 0.67 (sensitivity: 1.00, specificity: 0.95) and LVes EI ≥ 1.06 (sensitivity: 1.00, specificity: 0.97).

**Conclusion:**

We provide normal echocardiographic reference values of the RV/LVes ratio and LVes EI in healthy children, as well as statistically determined cutoffs for the increased values in children with PH.

## Introduction

Right ventricular (RV) and left ventricular (LV) size and function are important determinants for diagnosis, treatment and follow-up in a number of pediatric cardiovascular conditions, including pulmonary hypertension (PH) and congenital heart disease (CHD) (1–7). Because of growth throughout childhood, interpretation of these measurements requires normalization of the according variables to the size of the body. In pediatric PH research, awareness of the importance of biventricular size and function variables and ventricular-ventricular interaction (VVI) is increasing (8–16). The impact of the RV “under pressure” is also related to underfilling and dysfunction of the compressed left ventricle, which reduces LV output (13, 17). During direct interaction, the LV cavity is compressed owing to leftward bowing of the ventricular septum, causing impaired LV filling, and low cardiac output (8, 18). The echocardiographically derived RV/LV endsystolic ratio (RV/LVes ratio) and the LV endsystolic eccentricity index (LVes EI), both measured in the parasternal short axis view, are potentially useful diagnostic variables for patients with suspected PH. The RV/LVes ratio and LVes EI are the most important surrogates for disease severity when tricuspid regurgitation velocity jet is not interpretable (in about 30–54% of pediatric PH echocardiograms (15, 19–21).

The aim of the study was to determine normal reference values for RV/LVes ratios and LVes EI, and possible associations of these variables with age, body length (BL), body weight (BW), body surface area (BSA), and gender in a large healthy pediatric cohort, including neonates. This is the first study to provide data on representative, normal, pediatric RV/LVes ratios and LVes EI with according reference values and to compare such data with values obtained in a small but representative pediatric PH cohort. We hypothesized that higher RV/LVes ratios and LVes EI may become sufficient markers for the diagnosis and follow-up of children with PH when compared to normative values.

## Materials and Methods

### Primary Aim of the Study

We aim to establish RV/LVes ratios and LVes EI age specific normative values and reference values for healthy children (age: 1 day up to 18 years).

### Healthy Study Group

The study group consisted of 769 healthy children. The study subjects were recruited from healthy children referred to the Department of Pediatrics; Medical University Graz, Austria for evaluation of a heart murmur or a family history of heart disease.

#### Inclusion Criteria

Age from first day of life to 18 years, male or female, echocardiograms with an official reading of “normal cardiovascular anatomy and function.” Only subjects whose physical examination was judged as being normal were included.

#### Exclusion Criteria

Patients with an acquired heart diseases, chest and thoracic spine deformities, chromosomal syndromes, patients with paradoxical septum motion including patients with right bundle branch block, as well as patients with hemodynamically significant patent ductus arteriosus, significant ventricular septal defect or atrial septum defect, pulmonary artery stenosis or insufficiency were excluded. Patients were examined in a resting state.

For children of the “healthy cohort,” we confirmed a normal left ventricular ejection fraction, measured using the Simpsons formula, as well as a normal RV size (22) and RV systolic function (23) by including only patients with normal age-related RV size parameters compared to respective available normative values (5, 22, 24).

### Pulmonary Hypertension Study Group

The PH study group (Table 1) consisted of 44 children, including children with PH associated with congenital heart disease (PH-CHD) 48%, PH associated with bronchopulmonary dysplasia (PH-BPD) 41%, and patients with idiopathic pulmonary arterial hypertension (iPAH) 11%. The respective CHDs were surgically repaired in all patients at a mean age of 5.6 months (range: 0.6–15.3). All VSD, AVSD, and complex CHDs patients had a partial/complete bundle branch block after operation (varying from 0.10to 0.15 s QRS duration), while patients after ASD closure had narrow QRS. Patients with hemodynamic relevant conduit regurgitation or stenosis, right ventricular outflow tract obstruction, relevant intracardiac shunts, Fontan physiology, Eisenmenger Syndrome, active pacing, were excluded from the study. At time of enrolment, all patients were clinically stable without change of medications within the preceding 4 months.

**TABLE 1:** Demographic data of the children with pulmonary hypertension (PH).

Patients characteristics	
**All PH patients n or median [IQR]**	
Number (*n*)	44
Female (%)	32
Age years (range)	2.1 [0.5–6.6] (0.1–17.7)
Body weight kilogram (range)	10.4 [5.1–18.4] (1.5–47)
Body length centimeter (range)	80 [55–118] (40–163)
BSA (body surface area) m^2^ (range)	0.5 [0.27–0.77] (0.1–1.5)
**PH categories**	
PAH-CHD (group 1.4.4)	17 (39%)
IPAH (group 1.1)	5 (11%)
Complex PH-CHD (group 5.4)	4 (9%)
PH BPD (group 3.5 PH)	18 (41%)
**PH medication**	
None (treatment naïve)	2
Duale therapy: ERA* + PDE5	18
Monotherapy (PDE5 or ERA*)	24
Inhaled or intravenous PH therapy	0
**PH- CHD diagnosis**	
AVSD repair	5
VSD repair	3
PDA closure	3
ASD I repair	1
ASD II repair	5
Complex CHD	4
**NYHA FC**	
I	13
II	22
III	9
IV	0

Age of the patients at baseline is the age of inclusion into the study. PH, Pulmonary hypertension; PAH, Pulmonary arterial hypertension; PAH-CHD, PAH associated with congenital heart disease; PH, pulmonary hypertension; PH-BPD, PH due to bronchopulmonary dysplasia; IPAH, idiopathic PAH; Complex PH-CHD, Complex pulmonary hypertension associated with congenital heart disease; AVSD, atrioventricular septal defect; VSD, ventricular septum defect; PDA, patent ductus arteriosus; ASD, atrium septum defect; PDE5, Phosphodiesterase type 5; ERA, Endothelin receptor Antagonist. *Macitentan or Bosentan.

### Echo Protocol

Echocardiograms were performed using a commercially available echocardiographic system (Sonos iE33, Philips, Andover, Mass, United States) using transducers of 5–1, 8–3, and 12–4 MHz depending on patient age, size, and weight. Images were recorded digitally and analyzed using off-line software (Xcelera Echo; Philips Medical Systems, The Netherlands).

**FIGURE 1:**
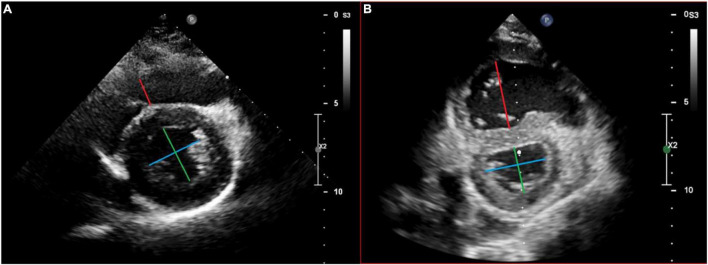
Echocardiographic measurement of the right ventricular to left the ventricular endsystolic (RV/LVes) ratio and the left ventricular endsystolic eccentricity index (LVes EI) in a healthy subject **(A)** and in a patient with pulmonary hypertension **(B)**. Measurement of the RV/LVes ratio (RV = red/LV = green line) and the LVes EI (blue/green line) in the apical parasternal short axis view between papillary muscle, from the endocardial to endocardial surfaces at endsystole.

The RV/LVes ratios were measured from the parasternal short axis (PSAX) view at mid LV level between papillary muscle, from the endocardial to endocardial surfaces at endsystole (Figures 1A,B) as described by Jone et al. (12). The LVes EI were calculated similar to the method, first described by Ryan et al. (25) as the ratio of LV diameter parallel to the septum divided by diameter perpendicular to the septum (Figures 1A,B). However, we measured both (RV/LVes ratio and LVes EI) between the papillary muscle levels. Es was defined similar to Burkett et al. (26) as the final frame of free-wall contraction, immediately prior to outward motion (relaxation) of the LV free-wall. In PH, systole and diastole between the RV and LV can occur at different times, with RV diastolic inflow significantly delayed beyond LV inflow due to prolonged systole and/or isovolumic relaxation; this delay is related to increased afterload (8). Thus, endsystole and end-diastole can be different for each ventricle in PH, leading to confusion (8). To simplify, we used the contractile pattern of the LV to define endsystole like Vonk Noordegraaf et al. (4). In early LV diastole (outward motion of the free-wall), while the RV is still under increased pressure due to PH, there is often more profound leftward septal displacement (14). To minimize variability, a strict institutional protocol for image acquisition was used for this study. Age, BW, BL, BSA, and non-invasive blood pressure were measured at time of echocardiography, and the BSA was calculated using the Mosteller formula (27).

### Additional Measurements for Pulmonary Hypertension Children

NYHA FC/modified ROSS score were determined by 2 independent pediatric cardiologists, who are responsible for the medical care of the patients. The tricuspid annular plane systolic excursion (TAPSE) reflects the longitudinal excursion of the tricuspid annulus toward the apex and was measured with M-mode in the apical 4-chamber view as described before (23). The pulmonary artery acceleration time (PAAT) was measured as the interval between the onset of ejection and the peak flow velocity, defined as the time from the onset to maximal velocity (28) and is an inverse surrogate of pulmonary artery pressure and pulmonary vascular resistance index (PVRi).

### Ethics

This study complies with the institutional guidelines related to patient confidentiality and research ethics including the institutional review board approval of the Ethics Board of Graz Medical University (Ethics committee Number 33–320 ex 20/21.).

### Statistics

**FIGURE 2:**
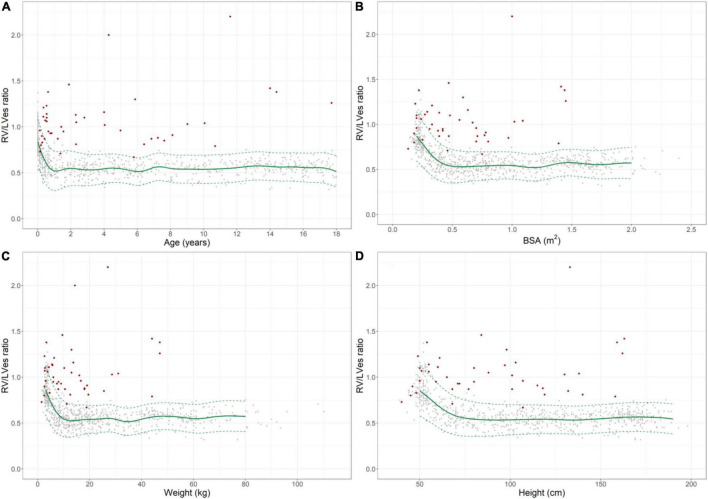
Normal reference values for right ventricular to left ventricular endsystolic (RV/LVes) ratio in healthy children and individual levels of pulmonary hypertension patients (red diamonds). Normal change of RV/LVes ratio with increasing **(A)** age, **(B)** BSA, **(C)** weight and **(D)** height (gray circles: individual values; green solid line: median, dashed green line: 2.5 and 97.5% percentile).

**FIGURE 3:**
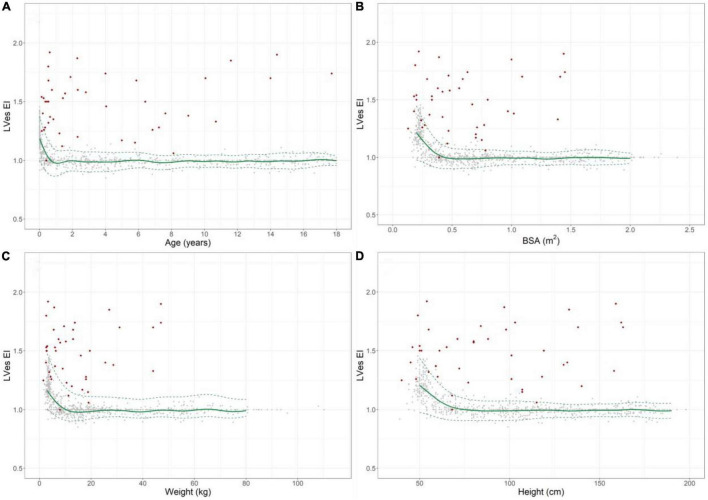
Normal reference values for LVes EI (LV endsystolic eccentricity index) in healthy children and individual levels of pulmonary hypertension patients (red diamonds). Normal change of LVes EI with increasing **(A)** age, **(B)** BSA, **(C)** weight, and **(D)** height (gray circles: individual values; green solid line: median, dashed green line: 2.5 and 97.5% percentile).

Data are presented as median and range (minimum–maximum) for continuous variables or absolute and relative numbers for categorical data. Associations of RV/LVes ratio and LVes EI with age, body length, weight, BSA, and clinical parameters were analyzed using Spearman’s rank correlation coefficient. Differences in RV/LVes ratio and LVes EI values between patients of different NYHA classes were analyzed using Kruskal Wallis test (Dunn’s *post hoc* test with the Bonferroni correction). To determine age-, body length-, weight-, and BSA-specific reference values, generalized additive models were used. Therefore, growth curve centile estimations were performed using either normal distribution or Reverse Gumbel distribution for the response variable. The decision regarding whether to use normal or Reverse Gumbel distribution was based on residual distribution. To define a “normal” range between 2.5 and 97.5% percentiles, the data are given in Supplementary Tables 3A–D, 4A–D and Figures 2A–D, 3A–D. For children older than 1 year, BSA > 0.5 m^2^, body length > 75 cm or weight > 15 kg, ROC analysis was performed to calculate AUC with 95% confidence intervals and the best cutoff to identify PH-patients. The best cutoff was defined by Youden-Index. For data analysis R (Version 4.1.1; R Foundation for Statistical Computing) was used. Interobserver and intraobserver variability was examined with an intraclass correlation coefficient (ICC) and corresponding 95% confidence intervals (95%CI).

## Results

### Healthy Children

In the 769 healthy neonates, children, and adolescents studied, no difference between males (50.7%) and females was observed in baseline characteristics (Supplementary Table 1).

### Right Ventricular to Left Ventricular Endsystolic Ratio

The RV/LVes ratio values were highly age dependent: Healthy neonates [median 0.83 (range 0.53–1.37)], healthy 12–24 months old [0.55 (0.35–0.80)], 18th year of life [0.53 (0.32–0.74)] (Figure 2A and Supplementary Table 2).

The RV/LVes ratio was high in healthy neonates 0.83 (range: 0.53–1.37) and gradually decreased within the first year of life (Figure 2A) and until increases in BSA (Figure 2B) to about 0.5 m^2^, body weight to about 15 kg (Figure 2C), and BL to about 75 cm (Figure 2D). The RV/LVes ratio was almost constant thereafter and did not further change with increasing age, BSA, body length and weight (18th year of life: median: 0.53, range: 0.32–0.74). The RV/LVes ratio negatively correlated with age (*p* < 0.001, r_*s*_ = –0.498;*p* < 0.001, r_*s*_ = –0.744 in infants < 1 year), BSA (*p* < 0.001, r_*s*_ = –0.484; *p* < 0.001, r_*s*_ = –0.740 in children with BSA < 0.5 m^2^), BW (*p* < 0.001, r_*s*_ = –0.484; *p* < 0.001, r_*s*_ = –0.764 in children with BW < 15 kg) and BL (*p* < 0.001, r_*s*_ = –0.486; *p* < 0.001, r_*s*_ = –0.701 in children with BL < 75 cm) (Figures 2A–D). Age-, BSA-, BL-, and BW-related normal RV/LVes ratio values are shown in Supplementary Tables 3A–D).

### Left Ventricular Endsystolic Eccentricity Index

LVes EI values were highly age dependent: Healthy neonates: [median 1.21 (range 0.92–1.45)], healthy 12–24 months old [1.0 (0.88–1.13)], 18th year of life: ratio: [1.0 (0.97–1.07)], Figure 3A and Supplementary Table 2.

The LVes EI was high in healthy neonates (median: 1.21, range: 0.92–1.45, Supplementary Table 2) and gradually decreased within the first year of life (Figure 3A) and until increases in BSA (Figure 3B) to about 0.5 m^2^, body weight to about 15 kg (Figure 3C) and BL to about 75 cm (Figure 3D), but were almost constant thereafter and did not further change with increasing age, BSA, body length, and weight (18th year of life: median: 1.00, range: 0.97–1.07), Figures 3A–D. The LVes EI negatively correlated with age (*p* < 0.001, r_*s*_ = –0.499; *p* < 0.001, r_*s*_ = –0.758 in infants < 1 year), BSA (*p* < 0.001, r_*s*_ = -0.493; *p* < 0.001, r_*s*_ = –0.696 in children with BSA < 0.5 m^2^), BW (*p* < 0.001, r_*s*_ = –0.493; *p* < 0.001, r_*s*_ = –0.722 in children with BW < 15 kg) and BL (*p* < 0.001, r_*s*_ = –0.494; *p* < 0.001, r_*s*_ = –0.642 in children with BL < 75 cm). Age-, BSA-, BL-, and BW-related normal LVes EI values are shown in Supplementary Tables 4A–D).

### Pulmonary Hypertension Children

**FIGURE 4:**
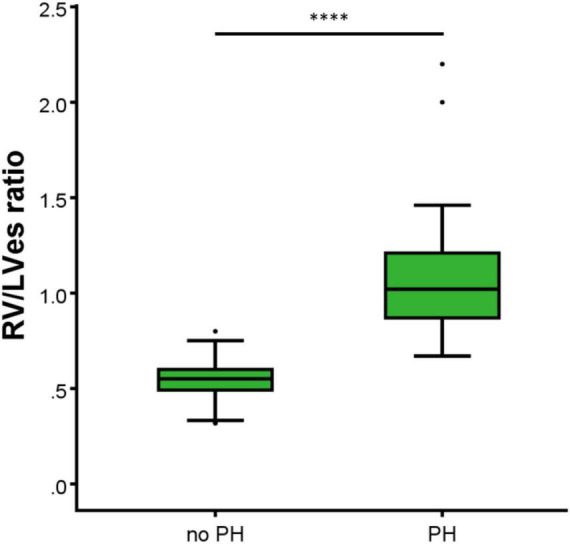
Differences between the healthy study group (no PH) and pulmonary hypertension (PH) study group in RV/LVes ratio (right ventricular to left ventricular endsystolic ratio). The box plot graphs show the median, IQR and range (Tukey method 1.5*IQR). ^****^p < 0.0001.

**FIGURE 5:**
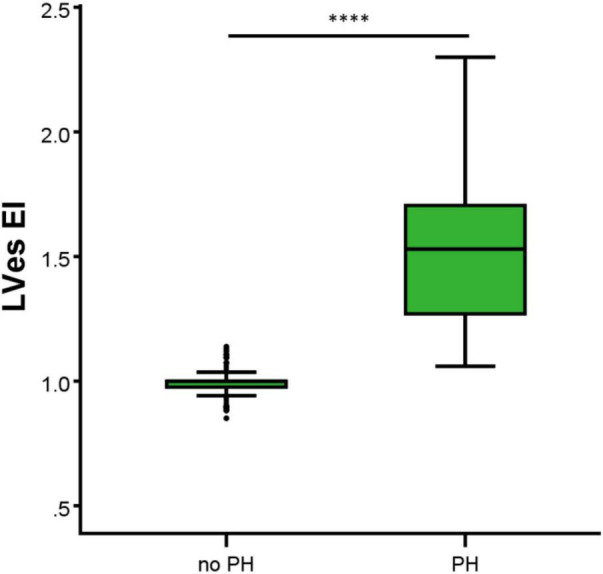
Differences between the healthy study group (no PH) and pulmonary hypertension (PH) study group in LVes EI (left ventricular endsystolic eccentricity index). The box plot graphs show the median, IQR and range (Tukey method 1.5*IQR). ^****^p < 0.0001.

In the 44 PH patients studied (median age: 2.1 years; range: 0.1 months to 17.7 years; 32% female) the RV/LVes ratio (median: 1.01, range: 0.67–2.20) and the LVes EI values (1.50, 1.06–2.30) did not change with age (RV/LVes: *p* = 0.257, r_*s*_ = 0.18; LVes EI *p* = 0.229, r_*s*_ = 0.19), body length (RV/LVes: *p* = 0.530, r_*s*_ = 0.10; LVes EI *p* = 0.544, r_*s*_ = 0.10), body weight (RV/LVes: *p* = 0.734, r_*s*_ = 0.05; LVes EI *p* = 0.799, r_*s*_ = 0.04), or BSA (RV/LVes: *p* = 0.732, r_*s*_ = 0.05; LVes EI *p* = 0.781, r_*s*_ = 0.04). Children (> 1 year) and adolescents with PH had significantly higher RV/LVes ratios (no PH: median 0.55, IQR 0.49–0.60; PH: 1.02, 0.87–1.26; *p* < 0.001) and higher LVes EI values (no PH: 1.00, 0.98–1.00; PH: 1.53, 1.26–1.71; *p* < 0.001) compared to children and adolescents without PH (Figures 4, 5).

**FIGURE 6:**
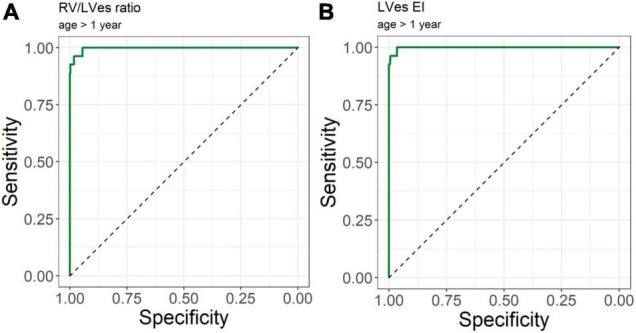
Receiver operating curve (ROC) analysis for the detection of pulmonary hypertension patients. Using **(A)** RV/LVes Ratio (AUC = area under the curve: 0.997, 95%CI: 0.993–1.000) and **(B)** LVes EI (AUC: 0.999, 95% CI: 0.996–1.000) in children older 1 year.

No difference between males and females were observed in LVes EI (median, range: female: 1.37, 1.06–1.87; male: 1.53, 1.15–2.30; *p* = 0.198) or RV/LVes ratio (female: 0.90, 0.67–2.20; male: 1.02, 0.81–2.00; *p* = 0.147). The best cutoff to differentiate children with PH from children without PH for RV/LVes ratio (Table 2) is ≥ 0.67 in children older than 1 year, > 0.5 m^2^ or > 75 cm (sensitivity: 1.00, specificity: 0.95, Figure 6A), for children with a weight of > 15 kg the best cutoff is ≥ 0.78 (sensitivity: 0.96, specificity: 1.00). For LVes EI the best cutoff (Table 2) is ≥ 1.06 for children older than 1 year, > 0.5 m^2^, > 75 cm (sensitivity: 1.00, specificity: 0.97) or > 15 kg (sensitivity: 1.00, specificity: 0.96, Figure 6B). In our cohort with a prevalence of 5.2% for RV/LVes ratio the positive predictive value was 50% and the negative predictive value 100% and for LVes EI the positive predictive value was 61.4% and the negative predictive value 100%. The RV/LVes ratio values (Supplementary Table 7) in the PH group showed a significantly negative correlation with PAAT, but no correlation was found between PAAT and LVes EI (Supplementary Table 8).

**TABLE 2:** Differentiation of children with pulmonary hypertension (PH) from children without PH.

	esRV/LV ratio				LVesEI			
	AUC (95%CI)	Best cutoff	Sens.	Spec.	AUC (95%CI)	Best cutoff	Sens.	Spec.
Age > 1 year	0.997 (0.993–1.000)	≥0.67	1.000	0.946	0.999 (0.996–1.000)	≥1.06	1.000	0.966
BSA > 0.5 m^2^	0.997 (0.992–1.000)	≥0.67	1.000	0.949	0.998 (0.995–1.000)	≥1.06	1.000	0.966
Body length > 75 cm	0.996 (0.989–1.000)	≥0.67	1.000	0.947	0.998 (0.993–1.000)	≥1.06	1.000	0.967
Body weight > 15 kg	0.997 (0.993–1.000)	≥0.78	0.958	0.996	0.998 (0.995–1.000)	≥1.06	1.000	0.962

*For children older than 1 year, BSA, body surface area; > 0.5 m^2^, BL, body length; > 75 cm or BW, Body weight; > 15 kg, ROC analysis was performed to calculate area under the curve (AUC) with 95% confidence intervals and the best cutoff to identify PH-patients. RV/LVes, Right ventricular to left the ventricular endsystolic ratio. LVes EI, left ventricular endsystolic eccentricity index. Sens, sensitivity; Spec, specificity.*

There was no correlation between TAPSE and RV/LVes ratio (Supplementary Table 7) or TAPSE and LVes EI (Supplementary Table 8). The RV/LVes ratio was significantly lower in patients with NYHA class 1 compared to class 2 or class 3 (class 1: 0.88, 0.67–1.09; class 2: 1.09, 0.71–1.46; *p* = 0.035; class 3: 1.10, 0.73–2.20). Patients with NYHA class 1 had significantly lower LVes EI values compared to class 2 (class 1: 1.28, 1.06–1.60; class 2: 1.52, 1.12–1.92; *p* = 0.039) and class 3 (1.68, 1.25–2.30; *p* = 0.009).

### Reproducibility

Interobserver and intraobserver variability was examined with an intraclass correlation coefficient (ICC) and corresponding 95% confidence intervals (95%CI).

Intraobserver ICC for the healthy LVes EI and RV/LVes ratio were as follows: ICC: 0.93, 95%CI: 0.88–0.96; ICC: 0.98, 95%CI: 0.96–0.99.

Interobserver ICC for the healthy LVes EI and RV/LVes ratio were as follows: 0.81, 95%CI: 0.70–0.88; ICC: 0.95, 95%CI: 0.92–0.97.

Intraobserver ICC for the PH LVes EI and RV/LVes ratio were as follows: ICC: 0.98, 95%CI: 0.97–0.99; ICC: 0.99, 95%CI: 0.99–1.00.

Interobserver ICC for the PH LVes EI and RV/LVes ratio were as follows: ICC: 0.97, 95%CI: 0.94–0.98, ICC: 0.96, 95%CI: 0.93–0.98.

## Discussion

We provide Age-, BSA-, BL-, and BW-related normal RV/LVes ratio (Figures 2A–D) and LVes EI (Figures 3A–D) reference values and percentiles (2.5, 50, and 97.5% percentile, Supplementary Tables 3A–D, 4A–D) in a large cohort of healthy neonates, children, and adolescents, as well as validated abnormal values in children with PH (median age: 2.1 years; range: 0.1 months to 17.7 years).

The physiology of ventricular-ventricular interactions is based on the in-series circulation and common pericardium, interventricular septum, and myocardial tracts running between the ventricles (29). A potential LV compression can be visualized by parasternal echocardiography in the short axis as a D-shaped LV, most simply quantified with the LV eccentricity index (EI) (25). This index measures the LV lateral dimension as a ratio over the anterior-posterior dimension in the parasternal short axis. In adults a LV EI > 1.1 is considered abnormal (25, 30). Ryan et al. (25) and Lammers et al. (31) demonstrated that children with PH have a more prominent systolic bowing of the interventricular septum than in diastole in their LV EI. The LV EI measures the deformity in the left ventricle, but does not take the altered RV dimension into account. The RV/LV ratio is measured from a parasternal short axis image at the LV papillary muscle level as the ratio of the anterior-posterior dimension of the RV- and LV diameters at end-systole (12, 26) or at end-diastole (26, 32, 33). The RV/LV ratio was shown to correlate with invasive measurements of PH (12, 26) and incorporates leftward septal shift and RV dilatation, thereby incorporating RV failure, remodeling, and adverse hemodynamics. An RV/LV ratio > 1 has been associated with increased mortality risk and thus is clinically useful (12).

In 1992 Louie et al. (34) reported of 11 adult PH patients (age 33 ± 10 years) with a LVes EI of 1.64 ± 0.48 and 11 healthy controls with a LVes EI of 1 ± 0.05. Averin et al. (21) reported of 29 PH subjects [3.8 years (0.9, 11.5), 72% IPAH] with a mean LV EI of 1.6 ± 0.5. Burkett et al. (26) reported of 78 healthy children/young adults (0–23 years) with a normal LVes EI of 1 (0.97–1.04) vs. 1.27 (1.1–1.5) in 78 PH patients (0–23 years; 92.3% under PH medication) investigated. We found that children (> 1 year) and adolescents with PH had significantly higher LVes EI values (no PH: median 1.00, IQR 0.98–1.00; PH: 1.53, 1.26–1.71) compared to children and adolescents without PH. In our study, healthy neonates had high LVes EI (median: 1.21, range: 0.92–1.45) and the RV/LVes ratio (median:0.83, range:0.53–1.37), both of which gradually decreased within the first year of life (Figures 2A, 3A) and until increases in BSA to about 0.5 m^2^ (Figures 2B, 3B), BW to about 15 kg (Figures 2C, 3C) and BL to about 75 cm (Figures 2D, 3D), but were almost constant thereafter and did not further change with increasing age, BSA, BW, and BL (LVes EI: 18 year of life: median: 1.00, range: 0.97–1.07; RV/LVes ratio: median: 0.53, range: 0.32–0.74).

The RV/LVes ratio and LVes EI values decreased significantly (*p* < 0.001) with increasing age, body weight, body length, and BSA in a non-linear way in the healthy patients. This highlights the importance of creating normal values for the pediatric age group, since indexed measurements to age, BSA, BL, and BW—due to the high variability of age-dependent growth—are highly warranted in this particular population. In contrast to toddlers, children, and adolescents with PH, the LVes EI and the RV/LVes ratio values were within the normative values in a substantial proportion of infants with PH. Therefore, the LVes EI and the RV/LVes ratio data in neonates and young infants should be interpreted with caution, due to the high variability in body size, weight, and pulmonary blood flow in this particular age group. While this phenomenon was not investigated within the last 30 years, Ichida et al. (35) reported in 1988 that the RV diameter in diastole is relatively large on the 1st day of life in healthy children (parasternal long axis RV: 1.05 ± 0.08) and the diastolic RV/LV ratio was found to be high (0.61 ± 0.05). While their data cannot be directly compared to our data (we investigated the ratio and LVes EI in the PSAX and not in the PLAX and at endsystole but not diastole), they give us a hint that this observed phenomenon is quite physiologic. Ichida et al. further found that the RV/LV ratio decreases gradually until 3 months of life, as well as the diastolic RV dimension. After the first months of life, a steady increase in RV dimension related to age is observed, while in contrast, the LV dimension was found to be gradually increased with age (35). Therefore, echocardiographic determination of the RV/LVes ratio and the LVes EI represents a promising approach for monitoring children with PH during standard echocardiographic examinations. The LVes EI turned out to be a useful variable of RV mechanics in infants with bronchopulmonary dysplasia and may be incorporated in routine protocols when there is a concern for PH in infants (19).

We (10) recently found that pediatric PH patients with increased LV EI values have significantly smaller LA areas. In the same study we found that the LV EI increased with an increasing systolic pulmonary artery pressure/systolic systemic arterial pressure ratio (sPAP/sSAP ratio) and also with an increasing pulmonary vascular resistance index, showing that the degree of LV compression due to significant RV afterload can be visualized. In a pediatric PH cohort, the RV/LV ratio was found to be positively correlated with an increasing sPAP/sSAP ratio and with an increasing NYHA FC (10). This suggests that these VVI variables (RV/LV ratio and the LV EI) potentially reflect the disease severity in children suffering from PH (10). Hansmann et al. (36) reported that the RV/LVes ratio and the LVes EI were greatly abnormal pre-lung transplantation (LuTx), with median values of 2.6 and 2.0, respectively. In contrast to the RV end-diastolic diameter that showed similar relative diameter reduction post-LuTx in most patients, the RV/LVes ratio and LVes EI had a “hand fan” type reduction pattern: Even PAH patients with very high ratios (RV dilation, LV compression/underfilling) pre-LuTx rapidly normalized both indices post-LuTx to indices of 0.62 and 1.0, respectively. These data demonstrate that readily available transthoracic echocardiography (2, 37) can sufficiently document the post-LuTx improvements of RV-hypertrophy, RV dilation, and LV filling. In this regard, the RV/LVes ratio (10, 38), the LVes EI (26, 39), and RV endsystolic remodeling index (9) are robust and useful echocardiographic markers (2, 11, 37, 38). Moreover, the LV EI and RV/LV dimension ratio were associated with a lower WHO functional class, and worse hemodynamics (26). This is in agreement with our findings; children with NYHA class 1 had significantly lower LVes EI and RV/LVes ratio values compared to class 2 and class 3.

Burkett et al. (26) showed that the systolic-, diastolic-, and maximum LV EI and the endsystolic and end-diastolic RV/LV ratio correlated with invasive hemodynamics (e.g., mean pulmonary arterial pressure, mPAP). An LVes EI of 1.16 yielded the largest AUC to define PH (LVes EI PH: 1.27 [1.1–1.49] vs. healthy subjects: 0.99 [0.97–1.04]). Only LVes EI and RV/LVes ratio correlated with the need to escalate therapy (26) suggesting that these VVI variables might be able to reflect the disease severity of PH also in children suffering from PH (26). Jone et al. (12) investigated the RV/LVes ratio retrospectively in 80 matched normal controls and 84 PH patients without shunts. Of their PH patients, 49 children underwent 94 echocardiographic examinations and cardiac catheterizations within 48 h (13 patients had simultaneous measurements). The RV/LVes ratio was correlated with hemodynamic variables. Twenty-two PH patients with RV/LV ratios > 1 had adverse events within a median of 1.1 years from their earliest echocardiographic studies. Increasing RV/LV ratio was associated with an increasing hazard for a clinical event. The RV/LVes ratios were lower in controls compared to patients with PH (mean, 0.51 [95% confidence interval, 0.48–0.54] vs. 1.47 [95% confidence interval, 1.25–1.70]), (12). We observed that children (> 1 year) and adolescents with PH had significantly higher RV/LVes ratio (no PH: median 0.55, IQR 0.49–0.60; PH: 1.02, 0.87–1.26; Figure 4) and higher LVes EI values (no PH: 1.00, 0.98–1.00; PH: 1.53, 1.26–1.71; Figure 5) compared to children and adolescents without PH. In children older than 1 year, the best cutoff to differentiate children with PH from children without PH for RV/LVes ratio was ≥ 0.67 (sensitivity: 1.00, specificity: 0.95) and for LVes EI ≥ 1.06 (sensitivity: 1.00, specificity: 0.97), Table 2 and Figure 6. The RV/LVes ratio values (Supplementary Table 7) in the PH group showed a significantly negative correlation with PAAT, but no correlation was found between PAAT and LVes EI (Supplementary Table 8). There was no correlation between TAPSE and RV/LVes ratio (Supplementary Table 7) or between TAPSE and LVes EI (Supplementary Table 8).

## Conclusion

Taken together, we provide normal reference values and percentiles (of the RV/LVes ratio and LVes EI in healthy children and adolescents (1 day–18 years) that will assist to identify children with abnormal values. The RV/LVes ratio and the LVes EI have been shown to be useful, simple, reliable, and non-invasive markers of right ventricular hypertension in infants with PH (11, 12, 19, 36, 40) and may be incorporated in routine protocols when there is a concern for PH in children.

### Study Limitations

The LV EI has limited power in postoperative CHD-PH patients due to an unpredictable effect of, e.g., a VSD repair on the long-time performance of the interventricular septum. In our postoperative CHD-PH patients, the effect of a bundle branch block on the dimensions (LVes EI and RV/LVes ratio) was not investigated. A bundle branch block may influence LVes EI and RV/LVes ratio. However, to what extent a bundle branch block may alter these values remains speculative. We do not provide more sophisticated indicators of RV remodeling and RV-LV interactions, such as 3D echocardiography.

## Data Availability Statement

The datasets presented in this article are not readily available. Requests to access the datasets should be directed to corresponding author.

## Ethics Statement

The studies involving human participants were reviewed and approved by the Ethics Board of Graz Medical University (Ethics committee Number 33–320 ex 20/21.). Written informed consent to participate in this study was provided by the participants or their legal guardian/next of kin.

## Author Contributions

SS, GH, and MK: conception and design and manuscript writing. SK-K, PC, and AG: administrative support. GG, AB, AF, and NN: provision of study materials or patients. SS and MK: collection and assembly of data. AA: data analysis and interpretation. All authors final approval of manuscript.

## Conflict of Interest

The authors declare that the research was conducted in the absence of any commercial or financial relationships that could be construed as a potential conflict of interest.

## Publisher’s Note

All claims expressed in this article are solely those of the authors and do not necessarily represent those of their affiliated organizations, or those of the publisher, the editors and the reviewers. Any product that may be evaluated in this article, or claim that may be made by its manufacturer, is not guaranteed or endorsed by the publisher.

## Abbreviations

BL: body lengths
BSA: body surface area
BW: body weight
CHD: congenital heart disease
EI: eccentricity index
Es: endsystolic
iPAH: idiopathic pulmonary arterial hypertension
LuTx: lung transplantation
LV: left ventricular
LVes EI: LV endsystolic eccentricity index
NYHA FC: New York Heart Association Functional class
PAAT: pulmonary artery acceleration time
PH: pulmonary hypertension
PH-BPD: PH associated with bronchopulmonary dysplasia
PH-CHD: PH associated with congenital heart disease
PSAX: parasternal short axis
RV: right ventricular
RV/LV ratio: right ventricular to left ventricular ratio
RV/LVes ratio: right ventricular to left ventricular endsystolic ratio
sPAP/sSAP ratio: systolic pulmonary artery pressure/systolic systemic arterial pressure ratio
TAPSE: tricuspid annular plane systolic excursion
VVI: ventricular-ventricular interaction.
