# Supplementary material for: Normal Echocardiographic Reference Values of the Right Ventricular to Left Ventricular Endsystolic Diameter Ratio and the Left Ventricular Endsystolic Eccentricity Index in Healthy Children and in Children With Pulmonary Hypertension
Source: Front Cardiovasc Med. 2022 Jul 14;9:950765. doi: 10.3389/fcvm.2022.950765 (PMC9332913; doi:10.3389/fcvm.2022.950765)
Supplement: Supplementary file 1 [file Data_Sheet_1.pdf]

Supplementary Material

TABLE OF CONTENTS

	page
**Abbreviations and Acronyms**	**2**
**Supplementary Tables**	**3-16**
Table S1	3
Table S2	4
Table S3A	5
Table S3B	6
Table S3C	7
Table S3D	8
Table S4A	9
Table S4B	10
Table S4C	11
Table S4D	12
Table S5	13
Table S6	14
Table S7	15
Table S8	16

ABBREVIATIONS AND ACRONYMS

BL=body lengths (BL)

BSA= body surface area

BW=body weight

CHD=congenital heart disease

Cm=centimeter

Cm^2=square centimeter

Kg= kilogram

LV=left ventricular

LVes EI=LV endsystolic eccentricity index

n= number

PAAT= pulmonary artery acceleration time

PH= pulmonary hypertension

RV= right ventricular

RV/LVes ratio= right ventricular to left ventricular endsystolic ratio

TAPSE = tricuspid annular plane systolic excursion

SUPPLEMENTARY TABLES

Table S1

	male (n=390) Median (min-max)	female (n = 379) Median (min-max)	significance
Body Weight (kg)	15.50 (2.10-110.20)	16.30 (1.70-96.00)	0.843
Body Length (cm)	99.10 (43.00-198.00)	104.50 (39.00-186.00)	0.722
BSA*(cm²)	0.65 (0.16-2.40)	0.69 (0.14-2.09)	0.796
Age (years)	3.35 (0.00-17.99)	3.69 (0.00-18.00)	0.439
LVes EI	1.00 (0.85-1.45)	1.00 (0.88-1.42)	0.671
RV/LVes ratio	0.59 (0.32-1.27)	0.58 (0.32-1.37)	0.700

Table S1
Demographic data: LV endsystolic eccentricity index (LVes EI) and right to left ventricular endsystolic ratio (RV/LVes ratio) of the healthy male and female study group.

Table S2

	Healthy neonates	Healthy: 18 years old
	Median (min–max)	Median (min–max)
Body Weight (kg)	3.40 (1.70-4.70)	61.00 (34.40-93.50)
Body Length (cm)	51.00 (39.00-62.00)	172.00 (154.00-198.00)
BSA* (cm^2)	0.22 (0.14-0.27)	1.71 (1.21-2.24)
Age (years)	0.011 (0.003-0.083)	17.48 (17.08-18.00)
LVes EI	1.21 (0.92-1.45)	1.00 (0.97-1.07)
RV/LVes ratio	0.83 (0.53-1.37)	0.53 (0.32-0.74)

Table S2
Demographic data: LV endsystolic eccentricity index (LVes EI) and right ventricular to left ventricular endsystolic ratio (RV/LVes ratio) of the healthy neonates and healthy 18 year old adolescents

Table S3A

RV/LVes ratio			
Age	2.5%	50%	97.5%
1 week	0.58	0.83	1.08
2 weeks	0.57	0.82	1.07
3 weeks	0.56	0.81	1.06
1 month	0.54	0.79	1.04
2 months	0.50	0.75	0.99
3 months	0.46	0.71	0.95
4 months	0.43	0.67	0.91
5 months	0.40	0.64	0.87
6 months	0.37	0.61	0.84
7 months	0.35	0.58	0.81
8 months	0.34	0.56	0.79
9 months	0.32	0.54	0.77
10 months	0.31	0.53	0.75
11 months	0.31	0.52	0.74
1 year	0.31	0.52	0.74
2 years	0.36	0.55	0.74
3 years	0.36	0.53	0.70
4 years	0.38	0.54	0.70
5 years	0.39	0.54	0.70
6 years	0.36	0.51	0.66
7 years	0.41	0.56	0.71
8 years	0.40	0.55	0.70
9 years	0.39	0.54	0.69
10 years	0.38	0.54	0.69
11 years	0.39	0.54	0.70
12 years	0.40	0.56	0.71
13 years	0.42	0.57	0.73
14 years	0.42	0.57	0.72
15 years	0.41	0.56	0.72
16 years	0.40	0.56	0.71
17 years	0.39	0.55	0.72
18 years	0.34	0.51	0.68

Table S3A
Normative (2.5, 50, 97.5% percentile) age related values of the right ventricular to left ventricular endsystolic ratio (RV/LVes ratio)

Table S3B

RV/LVes ratio			
BSA*	2.5%	50%	97.5%
0.200	0.60	0.87	1.14
0.225	0.56	0.83	1.09
0.250	0.53	0.78	1.04
0.275	0.50	0.74	0.99
0.30	0.46	0.70	0.94
0.35	0.41	0.63	0.85
0.4	0.37	0.58	0.79
0.5	0.35	0.53	0.72
0.6	0.36	0.53	0.70
0.7	0.37	0.54	0.70
0.8	0.38	0.54	0.69
0.9	0,39	0,54	0.70
1.0	0.39	0.54	0.69
1.1	0.38	0.53	0.68
1.2	0.37	0.52	0.67
1.3	0.39	0.54	0.69
1.4	0.42	0.57	0.72
1.5	0.43	0.58	0.72
1.6	0.41	0.56	0.71
1.7	0.40	0.55	0.71
1.8	0.40	0.56	0.72
1.9	0.40	0.57	0.73
2.0	0.40	0.57	0.74

Table S3B
Normative (2.5, 50, 97.5% percentile) BSA related values of the right ventricular to left ventricular endsystolic ratio (RV/LVes ratio).*Body surface area in m^2

Table S3C

RV/LVes ratio			
BL*	2.5%	50%	97.5%
50	0.58	0.85	1.13
55	0.52	0.78	1.03
60	0.45	0.69	0.93
65	0.40	0.62	0.84
70	0.37	0.58	0.79
75	0.36	0.55	0.75
80	0.36	0.54	0.73
85	0.36	0.54	0.72
90	0.36	0.53	0.71
95	0.36	0.53	0.70
100	0.37	0.54	0.70
110	0.38	0.54	0.70
120	0.39	0.54	0.70
130	0.38	0.53	0.69
140	0.38	0.53	0.69
150	0.39	0.55	0.70
160	0.40	0.56	0.72
170	0.41	0.57	0.72
180	0.40	0.56	0.72
190	0.38	0.54	0.71

Table S3C

Normative (2.5, 50, 97.5% percentile) BL related values of the right ventricular to left ventricular endsystolic ratio (RV/LVes ratio). *Body length in cm

Table S3D

RV/LVes ratio			
BW*	2.5%	50%	97.5%
3	0.59	0.86	1.13
4	0.54	0.79	1.05
5	0.48	0.73	0.97
6	0.44	0.67	0.90
7	0.41	0.63	0.85
8	0.38	0.59	0.80
9	0.36	0.56	0.77
10	0.35	0.55	0.74
11	0.35	0.53	0.72
12	0.34	0.53	0.71
13	0.35	0.52	0.70
14	0.35	0.53	0.70
15	0.36	0.53	0.70
16	0.37	0.53	0.70
17	0.37	0.54	0.70
18	0.38	0.54	0.70
19	0.38	0.54	0.69
20	0.39	0.54	0.69
25	0.40	0.55	0.70
30	0.39	0.54	0.69
35	0.37	0.51	0.66
40	0.39	0.54	0.69
45	0.42	0.57	0.72
50	0.43	0.57	0.72
55	0.41	0.56	0.71
60	0.39	0.55	0.70
65	0.39	0.56	0.72
70	0.41	0.57	0.74
75	0.41	0.58	0.74
80	0.41	0.57	0.74

Table S3D
Normative (2.5, 50, 97.5% percentile) BW related values of the right ventricular to left ventricular endsystolic ratio (RV/LVes ratio). *Body weight in kg

Table S4A

LVes EI			
Age	2.5%	50%	97.5%
1 week	0.99	1.18	1.37
2 weeks	0.99	1.17	1.36
3 weeks	0.98	1.17	1.35
1 month	0.97	1.16	1.34
2 months	0.95	1.13	1.30
3 months	0.93	1.10	1.26
4 months	0.92	1.07	1.23
5 months	0.90	1.05	1.20
6 months	0.89	1.03	1.17
7 months	0.88	1.02	1.15
8 months	0.87	1.00	1.13
9 months	0.87	0.99	1.12
10 months	0.87	0.98	1.10
11 months	0.87	0.98	1.09
1 year	0.87	0.98	1.09
2 years	0.91	1.00	1.08
3 years	0.90	0.99	1.07
4 years	0.90	0.99	1.07
5 years	0.92	0.99	1.07
6 years	0.94	1.00	1.07
7 years	0.92	0.98	1.05
8 years	0.93	0.98	1.04
9 years	0.94	1.00	1.05
10 years	0.94	0.99	1.04
11 years	0.94	0.99	1.05
12 years	0.94	0.99	1.04
13 years	0.94	0.99	1.05
14 years	0.93	0.99	1.05
15 years	0.93	0.99	1.06
16 years	0.93	1.00	1.06
17 years	0.95	1.01	1.06
18 years	0.96	1.00	1.04

Table S4A
Normative (2.5, 50, 97.5% percentile) age related values of the LV endsystolic eccentricity index (LVes EI)

Table S4B

LVes EI			
BSA*	2.5%	50%	97.5%
0.200	0.98	1.21	1.45
0.225	0.97	1.18	1.39
0.250	0.96	1.15	1.34
0.275	0.95	1.12	1.29
0.30	0.94	1.09	1.24
0.35	0.92	1.04	1.17
0.4	0.91	1.01	1.12
0.5	0.90	0.99	1.08
0.6	0.90	0.99	1.07
0.7	0.91	0.99	1.07
0.8	0.92	0.99	1.06
0.9	0.93	1.00	1.06
1.0	0.94	0.99	1.05
1.1	0.94	0.99	1.05
1.2	0.94	0.99	1.05
1.3	0.93	0.99	1.04
1.4	0.94	0.99	1.05
1.5	0.94	1.00	1.06
1.6	0.93	1.00	1.06
1.7	0.93	1.00	1.07
1.8	0.94	1.00	1.06
1.9	0.94	0.99	1.05
2.0	0.95	0.99	1.04

Table S4 B

Normative (2.5, 50, 97.5% percentile) BSA related values of the LV endsystolic eccentricity index (LVes EI). *Body surface area in m^2

Table S4C

LVes EI			
BL*	2.5%	50%	97.5%
50	0.98	1.20	1.43
55	0.96	1.14	1.33
60	0.93	1.08	1.24
65	0.91	1.04	1.16
70	0.91	1.01	1.12
75	0.90	1.00	1.09
80	0.91	0.99	1.08
85	0.90	0.99	1.07
90	0.90	0.99	1.07
95	0.90	0.99	1.07
100	0.91	0.99	1.07
110	0.92	0.99	1.07
120	0.93	0.99	1.06
130	0.93	0.99	1.05
140	0.94	0.99	1.04
150	0.94	0.99	1.05
160	0.94	0.99	1.05
170	0.94	1.00	1.06
180	0.93	0.99	1.06
190	0.93	0.99	1.05

Table S4C Normative (2.5, 50, 97.5% percentile) body length (BL) related values of the LV endsystolic eccentricity index (LVes EI).* Body length in cm

Table S4D

LVes EI			
BW*	2.5%	50%	97.5%
3	1.01	1.16	1.47
4	0.99	1.13	1.42
5	0.97	1.10	1.37
6	0.95	1.07	1.32
7	0.94	1.05	1.28
8	0.93	1.03	1.24
9	0.92	1.02	1.22
10	0.91	1.00	1.19
11	0.90	0.99	1.17
12	0.90	0.99	1.15
13	0.90	0.98	1.14
14	0.90	0.98	1.13
15	0.90	0.98	1.13
16	0.91	0.98	1.12
17	0.91	0.98	1.12
18	0.91	0.98	1.11
19	0.92	0.98	1.11
20	0.92	0.99	1.11
25	0.94	0.99	1.11
30	0.94	0.99	1.10
35	0.93	0.99	1.09
40	0.93	0.98	1.09
45	0.94	0.99	1.10
50	0.94	0.99	1.11
55	0.93	0.99	1.11
60	0.93	1.00	1.12
65	0.94	1.00	1.13
70	0.93	0.99	1.11
75	0.93	0.98	1.09
80	0.94	0.99	1.09

Table S4D

Normative (2.5, 50, 97.5% percentile) Body weight (BW) related values of the LV endsystolic eccentricity index (LVes EI). *Body weight in kg

Table S5

	Healthy neonates<br>Median (min –max)	PH: 18 years<br>Median (min –max)
Body Weight (kg)	3,40 (1,70-4,70)	61,00 (34,40-93,50)
Body Lenght (cm)	51,00 (39,00-62,00)	172,00 (154,00-198,00)
BSA*(cm²)	0,22 (0,14-0,27)	1,71 (1,21-2,24)
Age (years)	0,011 (0,003-0,083)	17,48 (17,08-18,00)
LVes EI	1,21 (0,92-1,45)	1,00 (0,97-1,07)
RV/LVes Ratio	0,83 (0,53-1,37)	0,53 (0,32-0,74)

Table S5

Demographic data: LV endsystolic eccentricity index (LVes EI) and and right ventricular to left ventricular endsystolic ratio (RV/LVes ratio) of the healthy neonates and 18 year old adolescents with PH. *body surface area.

Table S6

	N (%) Median (IQR)
male	390 (50.6%)
female	379 (49.2%)
Body Weight (kg)	15.8 (6.6-39.0)
Body Length (cm)	102.0 (64.0-146.5)
BSA*(cm^2)	0.67 (0.34-1.26)
Age (years)	3.36 (0.31-11.04)
LVes EI	1.00 (0.99-1.05)
RV/LVes ratio	0.58 (0.52-0.68)

Table S6
Demographic data, LV endsystolic eccentricity index (LVes EI) and right ventricular to left ventricular endsystolic ratio (RV/LVes ratio) of the healthy study group. *body surface area (BSA).

Table S7

		RV/LVes ratio
PAAT	Spearman's rho	-.306*
	P-val (2-tailed)	0.043
	N	44
TAPSE	Spearman's rho	-0.208
	P-val (2-tailed)	0.175
	N	44
LVes EI	Spearman's rho	.797**
	P-val (2-tailed)	<0.0001
	N	44

Table S7

Correlation of right ventricular to left ventricular endsystolic ratio (RV/LVes ratio) with echocardiographic parameters [Pulmonary Artery Acceleration Time (PAAT), tricuspid annular plane systolic excursion (TAPSE)] and LV endsystolic eccentricity index (LVes EI). *$p<0.05$, **$p < 0.0001$

Table S8

		LVes EI
PAAT	Spearman's rho	-0.261
	P-val (2-tailed)	0.090
	N	44
TAPSE	Spearman's rho	-0.239
	P-val (2-tailed)	0.123
	N	44
RV/LVes ratio	Spearman's rho	.783**
	P-val (2-tailed)	<0.0001
	N	44

Table S8

Correlation of LV endsystolic eccentricity index (LVes EI) with echocardiographic parameters (PAAT, TAPSE) and right to left ventricular endsystolic ratio (RV/LVes ratio. (**p < 0.0001).
